# Medical Imaging Biomarker Discovery and Integration Towards AI-Based Personalized Radiotherapy

**DOI:** 10.3389/fonc.2021.764665

**Published:** 2022-01-17

**Authors:** Yaru Pang, Hui Wang, He Li

**Affiliations:** ^1^ Department of Medical Physics and Biomedical Engineering, University College London, London, United Kingdom; ^2^ Department of Chemical Engineering, University College London, London, United Kingdom; ^3^ Department of Engineering, University of Cambridge, Cambridge, United Kingdom

**Keywords:** functional imaging, radiotherapy, personalized radiation dose, dose painting by contours, dose painting by numbers

## Abstract

Intensity-modulated radiation therapy (IMRT) has been used for high-accurate physical dose distribution sculpture and employed to modulate different dose levels into Gross Tumor Volume (GTV), Clinical Target Volume (CTV) and Planning Target Volume (PTV). GTV, CTV and PTV can be prescribed at different dose levels, however, there is an emphasis that their dose distributions need to be uniform, despite the fact that most types of tumour are heterogeneous. With traditional radiomics and artificial intelligence (AI) techniques, we can identify biological target volume from functional images against conventional GTV derived from anatomical imaging. Functional imaging, such as multi parameter MRI and PET can be used to implement dose painting, which allows us to achieve dose escalation by increasing doses in certain areas that are therapy-resistant in the GTV and reducing doses in less aggressive areas. In this review, we firstly discuss several quantitative functional imaging techniques including PET-CT and multi-parameter MRI. Furthermore, theoretical and experimental comparisons for dose painting by contours (DPBC) and dose painting by numbers (DPBN), along with outcome analysis after dose painting are provided. The state-of-the-art AI-based biomarker diagnosis techniques is reviewed. Finally, we conclude major challenges and future directions in AI-based biomarkers to improve cancer diagnosis and radiotherapy treatment.

## 1 Introduction

For treatment planning and outcome management, medical imaging plays a significant role to guarantee satisfying treatment of radiation therapy (RT) (1). Conventional anatomical imaging, such as CT and MRI, have been widely used to define the tumour size and locations. However, conventional anatomical imaging techniques provide limited insight into tumour macro- and micro-environments, especially regarding biological function, such as metabolic activity, cell proliferation, perfusion, hypoxia *etc.* This information can facilitate evaluating the severity of disease, improving tumour staging and thus the subsequent patient stratification and treatment (1). Therefore, interest in extracting quantifiable radiologic biomarkers with functional imaging has arisen recently (2). For example, functional images are used to protect critical-tissue and neural-nerve functionality during RT (2).

RT technology has been developed rapidly over the past decades and continues to be a standard treatment for malignant tumors, resulting in improved treatment outcomes over performing surgery or chemotherapy alone at certain cases. However, local recurrence after RT is one of the important modes of failure when dealing with most malignant tumors. The main reason may be that the planning target volume (PTV) of the tumor receives a uniform prescribed dose without considering the heterogeneity of the tumor itself in terms of time and space. In 1998, dose painting was originally proposed at ESTRO to address this challenge (3). In the 2000s, Ling et al. employed biological imaging to achieve “biological conformality”, where higher doses are applied to some areas with higher clonogenic cell density and radiation resistance in a tumour, while lower doses to less aggressive areas (3). In this way, tumor cells can be eliminated, and healthy tissues can recover faster (4, 5). To improve the accuracy of dose painting, many functional imaging optimisations have been proposed recently (2). Functional images have potential strengths to improve prognostication response to RT, which can facilitate personalized treatment and clinical trial designs in terms of patient-specific prescription dose and biological target volume (BTV) (6, 7). Moreover, quantitative functional imaging can be used for heterogeneous dose painting, where doses can be spatially redistributed throughout the target tumour based on personalized parameter maps (1).

Although current quantitative-imaging techniques are largely used for response management (8–11), there are only very limited studies on dose painting and no prior studies related to dose stratification. Therefore, clinical potential of quantitative function imaging becomes a prosperous direction that deserves investigations (1). Heide et al. suggested that high-quality imaging of the tumor and its surrounding tissues facilitate effective dose painting (2), and have discussed MRI-guided dose painting in 2012. In this article, we summarize various functional images as the pre-requisite for dose painting, as shown in the outline. The details of each image modality are discussed in Section 2. After that, dose painting by contours (DPBC) and dose painting by numbers (DPBN) using functional imaging are discussed in Section 3. Although at present, the supplementary exposure of dose painting to biological target areas is mostly implemented through PET-CT imaging technology, other modalities such as multi-parameter MRI also have potential in dose painting. In section 4, a review of recent AI-based biomarker diagnosis techniques is presented. On this basis, a biological target volume can be defined by identifying biomarkers from functional images. Section 5 concludes some existing challenges and provide corresponding possible research directions, including use of AI techniques in advanced functional imaging, though which we hope to inspire exciting developments and fruitful research avenues.

## 2 Functional Imaging

Because of the plethora of functional imaging techniques, this article focuses on DW-MRI, MRS, Perfusion MRI including Dynamic susceptibility contrast (DSC) and dynamic contrast enhanced (DCE), DTI MRI and PET-CT. We present a review for these methods by surveying the state-of-the-art works. **Table 1** summarised current functional imaging techniques and their biomarkers.

**Table 1:** An overview of functional imaging techniques.

Functional imaging techniques	Quantitative parameters	Biomarkers	Threshold
**MR-Spectroscopic (MRS)**	Metabolism	A ratio of choline to NAA (Cho/NAA)	Not clear
**Diffusion weighted (DW)-MRI**	Diffusion of water molecules	Apparent diffusion coefficient (ADC)	Not clear
**Perfusion MRI**	Tissue perfusion	Cerebral blood volume (CBV), cerebral blood flow (CBF), transfer constant of Gd- diethylenetriamine pentaacetic acid (Ktrans)	Relative (r) CBV > 1.75
**Diffusion tensor imaging (DTI)- MRI**	Tensor of water diffusion	White matter tracts (WMT)	Not clear
**PET-CT**	Glucose metabolism and the upregulation of glucose transporters in cancer cells	Standardized uptake value (SUV)	Not clear

### 2.1 DW-MRI

Diffusion-weighted magnetic resonance imaging (DW-MRI) is used to measure the mobility of water molecules in the microscopic environment of tissues. DW-MRI is very sensitive to cellular density, proliferation rate and cellular permeability (8, 9), and can reveal microscopic details of normal and diseased tissues. The sensitized signal is modelled by the amount of diffusion weights, called b-value. The amount of diffusion existing in the tissue, predominantly in the extracellular space (10, 11), is given by the apparent diffusion coefficient (ADC) (12). ADC map is an MRI image that shows better diffusion than conventional DWI (1). ADC map can be measured/defined by DW-MRI with at least two b-values (1). Darker areas in ADC images represent smaller magnitude of diffusion. Lower ADC values indicate slower water infusing where malignant tumour appears (13). Therefore, ADC and cellular density have an inverse correlation relationship. Several studies estimated the relationship between ADC and cellular density for different tumour sites. Ginat et al. performed a histological study and achieved the relationship between ADC and cellular density for chordoma (14), while Gupta et al. estimated the relationship for GBM (15).

**Figure 1:**
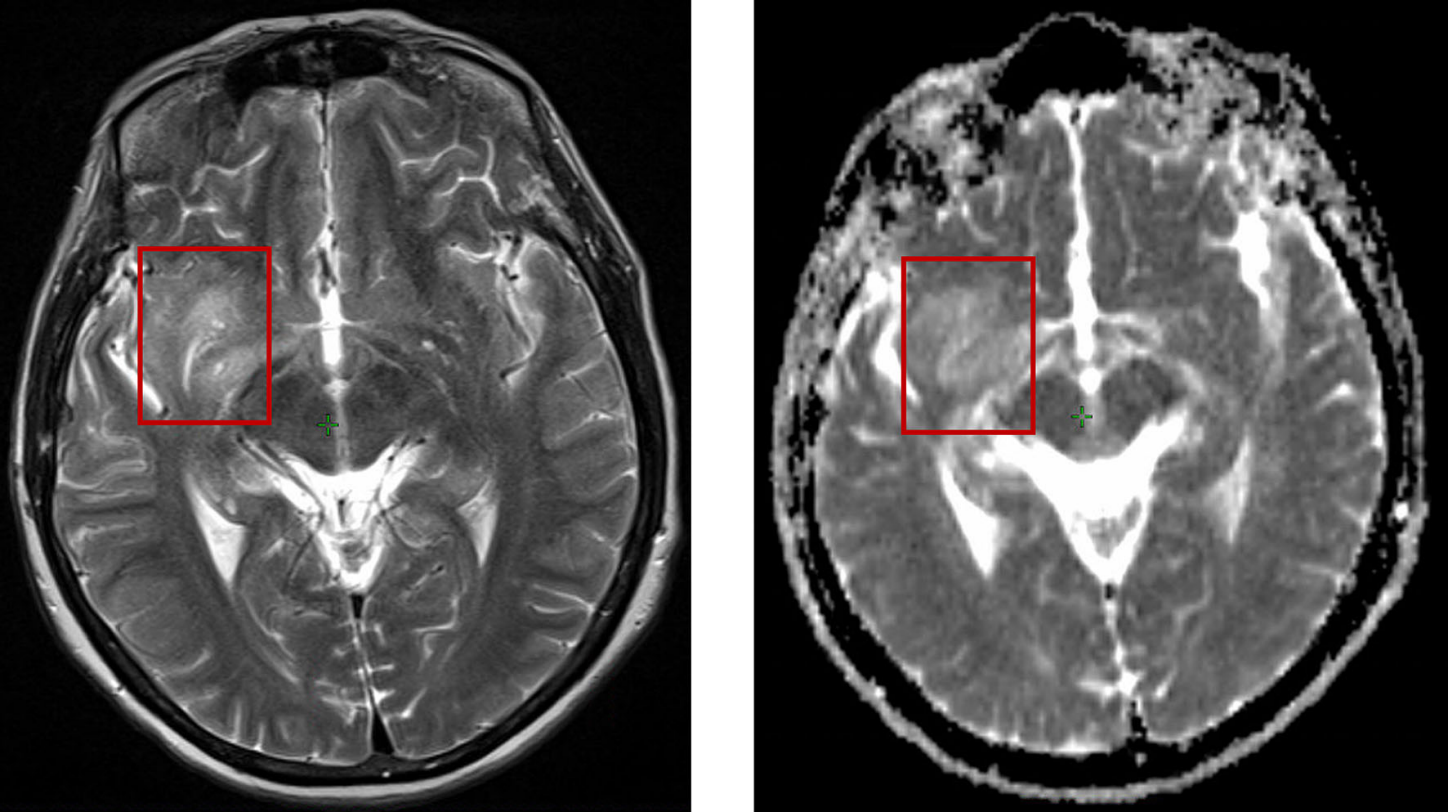
The same patient position shown in T2 weighted MRI (left) and DW-MRI (right), where T2 MRI represents more distinguishable classification than DW-MRI.

The restricted spread of water has been considered as an indicator of solid tumours. However, in DW-MRI with b values of 0 and 800-1000s/mm^2^, ADC values are usually unpredictable because of the mixture of micronecrosis, normal tissues, high-cell tumors and oedema, as shown in **Figure 1**. ADC values of a tumor are sometimes higher than those in normal tissues. Researchers used the minimum ADC value to determine its prognostic value (16–19). However, the area of malignant tumours is usually larger than the area with the minimum ADC value. Therefore, high b-value DW-MRI (e.g., 3000-4000s/mm^2^) are used to eliminate the signal from edema (20–22). Pramanik et al. showed that the hypercellular subvolume (HCV) of GBM determined by high-b value (3000s/mm^2^) DWI can predict progression-free survival (PFS) (23). About 40% of HCV may exceed the area of the traditional high-dose volume, which suggests a potential biological target with more radiation dose (23). Cao et al. showed an example of DW-MRI with two different b values. The lesions were more obvious in the high b-value DW-MRI (b=3000s/mm^2^) than the conventional b-value DW-MRI (b=800-1000s/mm^2^) (24).

Hamstra et al. (25) and Moffat et al. (26) provided information for individual patient’s adaptation to radiotherapy. Patients usually have higher overall survival (OS), whose voxel ADC values are changed dramatically after 3 weeks of radiotherapy (25, 26). However, when the tumor grows or shrinks, the paired images obtained before and during radiotherapy must consistently produce high-quality registration (27). ADC can be used to calculate tumour control probability (TCP) to analyse patient-specific characteristics. A recent study showed that MRI-driven cellular density can enhance TCP value differences in patients (28). Buizza et al. evaluated DW-MRI for modelling TCP in skull-base chordomas, which has enrolled in CIRT protocol (29). The aforementioned methods facilitate personalized and optimized treatments. The main limitation of these studies lies in uncertainties that inherently affect the relationship between ADC and cellular density. Moreover, single-shot echo planar imaging (EPI) pulse sequence is used in DW-MRI, which is very sensitive to geometric distortion. To reduce the geometric distortion due to EPI, multi-shot EPI, read-out segmented multi-shot EPI, and high parallel imaging factor are used. To achieve the golden standard, tumor target defined by high b-value DWI and parameter response curves therefore requires pathological verification.

### 2.2 MR-Spectroscopic Imaging

**Figure 2:**
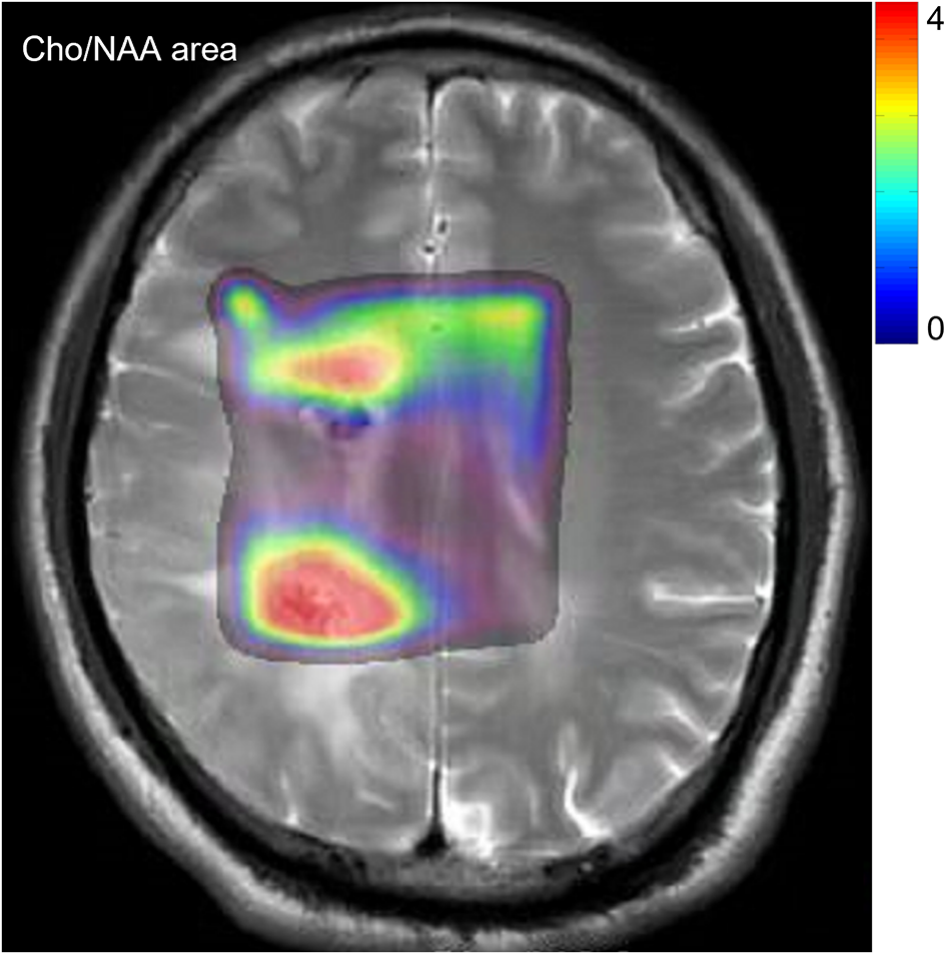
An example of MRS with a distribution of Choline/NAA.

MR-Spectroscopic Imaging (MRS) uses radio-labelled glucose and methionine to identify high-risk regions in large tumours. In MRS, active tumors exhibit at areas with high resonance in the choline spectral peak and a low NAA (N-acetylaspartate). **Figure 2** shows an example of MRS on post-operation MRI with choline/NAA. In other words, creatine resonance correlating with high choline/NAA, or choline/creatine ratios versus low ratios for areas of inactivity (30–37). Graves et al. analysed 36 patients with recurrent high-grade gliomas, who are treated with Gamma Knife stereotactic radiosurgery (SRS). Patients in high-risk regions of the SRS target had an improved survival rate versus those with MRS high-risk regions outside the SRS target (38). Croteau et al. studied 31 patients whose high-grade gliomas were resected after conventional MRI and MRS and found that MRS can accurately define the tumor boundary using histopathologic correlation (36). Moreover, Pirzkall et al. presented a pre-treatment analysis of 34 patients with high-grade gliomas (39) and found that high-risk regions defined by MRS were significantly smaller than regions in conventional T2-wighted imaging. Thanks to MRS, more normal areas in the brain can be distinguished from the tumour, thereby, reducing side effects (39).

Einstein et al. illustrated the procedure of Gamma Knife SRS incorporated with MRS functional imaging. Einstein et al. conducted the first prospective Phase II trial using MRS-targeted SRS for patients treated with GBM. In this work, the value of Cho/NAA> 2 was used as the area that is required to boost doses (40). However, the value of the Cho/NAA has no consensus to define the tumour area. The metabolic abnormality of Cho/NAA often exceeds the enhancement range of the lesion, and sometimes even exceeds the abnormal range of FLAIR (37, 41, 42). In some cases, the tumor recurs where the Cho/NAA is normal (41). Therefore, this technique has been discussed for several decades but not been transferred into clinical practice. Other obstacles include long acquisition, low spatial resolution, low robustness of spectral acquisition, and how to consistently obtain high-quality spectral images (1, 42, 43).

### 2.3 Perfusion MRI

Perfusion MRI is also a widely used method for tumour characterisation and diagnosis (1), which includes dynamic susceptibility contrast (DSC) and dynamic contrast enhanced (DCE) MRI. When using Perfusion MRI, patients will get injection of gadolinium-based agent during continuous image acquisition. Gadolinium contrast agents can decrease the T1 relaxation time. Therefore, the distribution within the patient can be studied by continuously acquiring T1-weighted images.

In particular for brain tumor, modelling the distribution of contrast agent in tissue allows us to quantify vascular leakage, cerebral blood volume (CBV), mean transit time and cerebral blood flow (CBF) (43–45). As tumor keeps growing, new blood vessels will appear. CBV, CBF and vascular leakage will be increased. Thus, CBV and CBF can be used as prognostic biomarkers to predict OS and progression-free survival PFS for tumours (43, 44, 46–48). Law et al. suggested that the mean relative CBV > 1.75 can be used as the threshold of low progression time for low grade and high grade GBM (43). As another biomarker, Gd-diethylenetriamine pentaacetic acid (Ktrans) can be used to quantify the leakage of vascular for OS prediction (49). Using the mean or median of CBF and CBV in the entire tumor volume has certain limitations for GBM, since it is a highly heterogeneous tumor which reduces the sensitivity of indicators for evaluating efficacy. Several methods have been proposed to solve this problem, such as dividing the entire tumor volume into several different sub-volumes according to different CBV values or vascular leakage (44, 46), and comparing the CBV before and during RT. Prior to the use of elevated CBV for defining enhancement target in GBM, the amount of elevated CBV in GBM related to tumors is a key factor. This makes sense since tumors can exist at areas where CBV is not elevated (50).

Even though DSC and DCE MRI can be used to estimate CBV, there are serval limitations of perfusion MRI. Similar with DW-MRI, DSC MRI has serious geometric distortion and signal loss because it is obtained by EPI. Moreover, CBV can be achieved from T1-weighted DCE MRI (51), however determining reliable arterial input function is still non-trivial. Use of a large sagittal view is able to determine the arterial input function of aorta, thereby reducing uncertainties. To incorporate CBV calculations into the RT workflow, a validated software is also necessitated. Last but not least, consider the fact that longer scanning time for every picture empowers large spatial resolution, however a high temporal resolution restricts the spatial resolution (52–54). Therefore, temporal and spatial resolution needs to be balanced.

### 2.4 Diffusion Tensor Imaging (DTI)-MRI

Diffusion tensor imaging (DTI)-MRI is one of the most popular technique to detect brain tumour and implement radiotherapy. Kelly et al. (55) and Price et al. (56) have shown that tumour cells preferably transmit along the white matter tracts (WMT) and have decreased infiltration in gray matter. Krishnan et al. investigated another retrospective study and showed the process from the original tumor to the recurrence using DTI-MRI for glioma patients. Their results confirmed that WMTs can be used as a route to indicate tumor spreading (57). These findings demonstrated that GBM grows along with WMTs. WMTs can be used as a biomarker to simulate tumor growth, and DTI-MRI can be used to define the corresponding CTV, as shown by serval retrospective studies (57–61). Moreover, DTI-MRI incorporated with mathematical model could define the RT target areas and evaluates the coverage rate of recurrence in patients with clinical target volume (CTV) derived from DTI.

Trip et al. implemented the first phase 0 study to deline CTVs using the DTI in post-operative chemo-/radiotherapy for GBM (61). Their results showed that CTVs derived by DTI-MRI performed worse for the central recurrences, however they are better for non-central and satellite recurrences. Unfortunately, this study lacks enough patient samples to conclude fair observations. In addition, the authors did not use the deformable registration, and measured the Hausdorff distance (HD) geometrically rather than anatomically. Nonetheless, due to the time-dependent migration of tumor cells, the effect of adopting a new target definition can only be truly evaluated in interventional studies.

### 2.5 Positron Emission Tomography (PET)-CT

Positron emission tomography (PET)-CT has been a valuable technique for RT in staging (62) and accurate target-volume delineation (63, 64). When imaging patients using PET-CT, a radioactive substance emitting positrons must be injected. Thereafter, the scanner detects the emitted photon pairs (511 keV) and quantifies their distribution throughout the patient after signal correction and normalization steps. A variety of PET radiotracers are available for *in vivo* imaging of biological processes. There are only five oncologic indications approved by the U.S. Food and Drug Administration, i.e. [^18^F]-fluoro deoxy-D-glucose (FDG), Na[^18^F], ^18^fluciclovine, [^11^C]-choline, and [^68^Ga]-DOTA-octreotate (DOTATATE). Many other candidates are being evaluated for clinical treatment.

As the most widely used PET radiotracer in the clinic, the details of FDG-PET are discussed in the following. FDG-PET depends on the relation between glucose metabolism and the upregulation of glucose transporters in cancer cells, and has played a significant role for patient staging, selection and RT target delineation (65–71). For example, mistreatment [^18^F]-FDG PET scan serves as a biomarker for adaptive dose painting. Kong et al. recently performed a phase II RT trial based on PET-CT for patients with NSCLC using interim [^18^F]-FDG PET to identify regions of poorly responding disease (72). An improved 2-year control rate was achieved with a factor of 82% versus 69% in the trial Radiation Therapy Oncology Group (RTOG) 1106 trial (73). Given the aforementioned results, FDG-PET is now the basis for RTOG 1106. Generally, the higher the uptake of FDG is, the more dose is needed, with a 10-30% increase to achieve the same control probability in the low FDG area (74).

Vogelius et al. derived dose–response functions for different structures that were distinguished by pre-treatment FDG-PET CT (75). Such dose response functions are used in the dose optimization process. Versus traditional treatments, they can maximize the patient’s TCP under the constraint of a constant average dose. Based on this study, Grönlund et al. implemented dose painting by numbers (DPBN) (76), and showed that TCP values were increased compared to uniformly delivered dose. Since uncertainties exist in the relationship between SUV and TCP, Grönlund et al. focused on the heterogeneity of SUV rather than absolute SUV, which provide a method to deal with the uncertain threshold of SUV.

## 3 Dose Painting

In recent decades, the advancement of radiotherapy has mainly benefited from advanced imaging technologies such as CT and MRI. Traditional tumor targets such as gross tumour volume (GTV) and clinical target volume (CTV) were defined in ESTRO-ACROP (77). Standard practice of radiotherapy is to give uniform radiation doses to the target area. However, this has been greatly challenged recently (78). Many factors affect the sensitivity of radiotherapy in the tumor, such as hypoxic area, cell proliferation rate, tumor cell density and intratumor blood perfusion, *etc.* (79). These factors change dynamically with the time and space during the treatment process (77). In view of the large heterogeneity of biological characteristics in tumors, when using uniform radiation dose for heterogeneous tumors. Therefore, it is possible to improve the local control by performing supplemental irradiation for the biological volumes that are relatively insensitive to treatment in the tumor. Given the aforementioned discussion in Section 2, modern biological imaging techniques, such as PET-CT, and multi-parameter MRI, can facilitate the development of dose painting (80). Dose painting is a new radiotherapy approach that produces optimized non-uniform dose distribution by using functional imaging for tumour control (80). Dose painting can be employed for three-dimensional (3D) radiobiological analysis, thereby investigating relations among relevant parameters in radiotherapy, the inherent potential to trace the real target volume, and therapeutic dose to control the disease. At present, the supplementary exposure of dose painting to biological target areas is mostly implemented through PET-CT imaging technology.

In order to deliver a relatively higher proportion of dose to a more resistant part of tumor, dose escalation and dose redistribution have been proposed recently. There are two main strategies of dose painting, dose painting by contours (DPBC) based on threshold of biomarkers and dose painting by numbers (DPBN) based on voxels. In DPBC, a tumour’s sub-volumes are heterogeneous in the functional images needed to be treated in a differentiated dose level (81). In DPBN, dose prescription is delivered to each voxel of a tumour, determined by the voxel value in functional images. Such voxel-based dose distribution is usually represented in a dose-prescription map (81). Their definitions, features and state-of-the-art advances are reviewed as follows. **Table 2** summarised the current DPBC and DPBN techniques and their application of dose escalation.

**Table 2:** A review of the state-of-the-art DPBC and DPBN techniques.

	Author	Year	Tumour place	Level of dose escalation	Conclusion
**DPBC**	Schimek-Jasch et al. (82)	2015	NSCLC	60-74 Gy	Target volume delineation is improved.
	Heukelom et al. (83)	2013	Head and neck	BR 77Gy, PTV outside the BR 67 Gy	5% improvement in LRC with a power of 80% at a significance level of 0.05.
	Kong et al. (84)	2013	NSCLC	84 Gy (median)	2-year rate of in-field LC and overall LC were 84%and 68%, the rate of OS was 51%.
	Fleckenstein et al. (85)	2011	NSCLC	66.6 to 73.8 Gy	Median survival time was 19.3 months.
	van Elmpt et al. (86)		NSCLC	BR 86.9 ± 14.9 Gy	Not Applicable
	Korreman et al. (87)	2010	NSCLC	90 Gy (mean)	Good conformity was obtained using MLC leaf width 2.5 mm, two arcs, and collimators 45/315 degrees, and robustness to positional error was low.
	Madani et al. (88)	2006	Head and neck	72.5, 77.5 Gy	Actuarial 1-year rates of LC were 85% and 87%, and 1-year rate of OS was 82% and 54% (P=0.06).
**DPBN**	Chen et al. (89)	2020	HNSCC	Not Applicable	Uncertainties in quantitative FDG-PET/CT imaging feedback arising from PVE and DIR have been analysed.
	Håkansson et al. (90)	2020	Head and neck	85.3 Gy(Maximum)	Proton dose-painting can reduce the non-target dose generally, but should avoid unintended hot spots of mucosal toxicity.
	Grönlund et al. (76, 91, 92)	2020,2019, 2017	Head and neck	CTVT 66 to 74.5 Gy	TCP values increased between 0.1% and 14.6% by the ideal doseredistributions for 59 patients.
	Jiménez-Ortega et al. (81)	2017	NSCLC	68 Gy (minimum)	The total planning time spent ranged from 6 to 8 h.
	Berwouts et al. (93)	2013	Head and neck	Prescription dose of GTV 70.2 Gy (median)	Disease control in 9/10 patients at a median follow-up of 13 months.
	Madani et al. (94)	2011	Head and neck	80.9 and 85.9 gy (median)	Actuarial 2-year rates of LC and freedom from distant metastasis were 95%, 93% and 68%, respectively.
	Meijer et al. (95)	2011	NSCLC	66 Gy	DPBN can increase higher dose levels than DPBC when considering organs at risk.

BR, boost region; CTVT, primary clinical target volume; LC, local-regional control; OS, overall survival; TCP, tumour control probabilities; GTV, gross tumour volume; HNSCC, squamous cell carcinoma of head and neck; PVE, partial volume effect; DIR, deformable image registration.

### 3.1 Dose Painting by Contours

In 2005, Ling et al. proposed the first DPBC technique (3). DPBC applies a dose boost by a certain threshold to a subvolume of the tumour. The regions of relatively lower and higher risk for recurrence are fixed with the threshold from the quantitative functional imaging. There are uncertainties related to the imaging modalities where the prescription function should be based, and when translating the image into a prescription function for dose painting (96, 97). Some major uncertainties, induced by PET imaging partial volume effect (PVE), tumour deformable image registration (DIR), and variation of the time interval between FDG injection and PET image acquisition have been investigated by Chen et al. (89).

In particular, interobserver variability in target volumes is a well-known factor for radiotherapy. For PET-CT based dose painting, detailed contouring guidelines can be referred to the PET-Plan (NCT00697333) clinical trial protocol (98). As a part of the PET-Plan quality assurance, a contouring dummy run (DR) was performed to analyse the interobserver variability (82). In addition, Korreman et al. investigated dose conformity by using the RapidArc optimizer and beam delivery technique (87). Optimisations for dose panting were performed in Eclipse by tuning the leaf width of multi-leaf collimator (MLC), the number of arc and collimator rotation. Positioning uncertainties are also considered for robust analysis and have been demonstrated with a low positional error (87).

A commonly used treatment planning approach for DPBC is the simultaneous integrated boost (SIB) technique (99) to increase the dose at a subvolume of the tumour. Patients treated by this approach are expected to achieve better dose tolerance (100). A homogeneous boost dose is assigned to the subvolume in many clinical trials (101). PET-guided DPBC was used to assess the feasibility of intensity-modulated radiotherapy (IMRT), where the maximum tolerated dose in head and neck cancer can be determined (88). The same purpose of increasing tolerated dose is also explored in non–small-cell lung cancer (NSCLC) treatments (85). Fleckenstein et al. proposed a source-to-background contouring algorithm for FDG-PET in the process of RT planning (85). There have been other advantages when using DPBC for RT planning. For example, Kong et al. demonstrated that adapting RT by boosting dose *via* DPBC to the FDG avid region improves 2-year local-regional tumour control and overall survival rate (84).

### 3.2 Dose Painting by Numbers

The term ‘‘dose painting by numbers” describes prescription dose on a voxel-by-voxel level (6). DPBN is a method to increase the additional dose gradually, adjusted by the local voxel intensities. Mathematical models are usually used to identify the relationship between the voxel values of the functional imaging and the risk of local recurrence (76). In particular, Chen et al. (89) analysed how the uncertainties in quantitative FDG-PET CT imaging impact intratumorally dose–response quantification, such as ones cause by PVE and tumour DIR (89). The negative effect arising from some of these uncertainties could be optimised by DPBN techniques (89).

There has been various technical feasibility and robustness of DPBN published recently (102–104). Dose prescription with steep gradients can be delivered by numerous subvolumes *via* a conventional linear accelerator (105). Rickhey et al. used the DPBN approach in brain tumours with [^18^F]-FET-PET, and achieved with high accuracy (106). Moreover, [^18^F]-FDG-PET-guided DPBN was proved to be feasible in phase I clinical trial by Berwouts et al. in head and neck RT (93). Recently, Grönlund et al. investigated the spatial relation between retrospectively observed recurrence volumes and pre-treatment SUV from FDG-PET (76). As were shown in the aforementioned studies, SUV driven dose–response functions have been presented to optimize ideal dose redistributions under the constraint of equal average dose of a tumour volume (76). A further analysis was proposed to investigate the feasibility of DPBN to increase the TCP in a clinical scenario (91).

Some DPBN approaches have been proposed by using sub-volumes as targets (76, 87, 91) or dose maps with prescription to the voxel as objective function (107), but these methods belong to dose-volume based optimization algorithms. Jiménez-Ortega et al. presented a new optimization algorithm to implement directly constraints to voxels instead of volumes, where Linear Programming (LP) is used to carry out DPBN approximation. This method is implemented in CARMEN, a Monte Carlo (MC) treatment planning system (81). Since proton therapy has been reported as potentially capable of decreasing toxicity, Håkansson et al. investigated DPBN in proton RT planning by comparing proton dose distributions with delivered photon plans from a phase-I trial of FDG-PET based dose-painting (90). Experimental results stay in line with the physical properties of the photon and proton beams, *i.e.* proton DPBN can be optimised with a quality comparable to photon DPBN (90).

### 3.3 Comparisons on DPBC and DPBN

DPBC mainly refers to the specific function image parameters to set the threshold for the replenishment area. Biomarkers in the high-risk area for recurrence have larger values over the defined threshold, while low-risk recurrence area corresponds to biomarkers having smaller values than the threshold. DPBN assumes that the recurrence risk of a certain pixel in the tumor area is positively correlated with the parameter intensity of its specific function image pixel, and the radiation dose of a certain pixel is directly related to its corresponding functional image pixel information. DPBN directly relies on theragnostic imaging (6). DPBN requires a customised software package to optimize the irradiation plan, but there is no commercial software directly implement optimization of DPBN (108, 109).

We remark that DPBC and DPBN have their own advantages and disadvantages (109). Advantages of DPBC are that sub volumes that need to boost dose can be pre-drawn before the treatment plan. Then the sub volumes can be set to add margins to supplement the geometric uncertainty, and the treatment plan can also be evaluated by conventional DVH. Disadvantages of DPBC mainly include the lack of consensus of the threshold for biomarkers. DPBN has more theoretical advantages than DPBC because it can deliver doses to voxel level. However, it cannot extend the margin of specific voxels and is more sensitive to uncertainty arising in image registration. Therefore, online image-guided treatment that can clearly show soft tissues is required.

Meijer et al. examined both DPBC and DPBN techniques for non-small cell lung cancer (NSCLC) patients’ treatment (95). In general, the amount of DPBC dose-boosting is limited whenever the GTV boost is close to any serial risk organ. However, DPBN shows significant higher dose values to high SUV voxels and are more distant from the organs at risk, since DPBN boosts work at a voxel-by-voxel basis (95).

## 4 AI-Based Biomarkers Diagnosis

We have investigated the potential of functional imaging on dose painting. With the help of the artificial intelligence (AI) techniques, we can define biological target volume by identifying biomarkers from functional images. Based on the degree of user interaction, methods used for the classification of various tumors can be classified as manual methods, semi-automatic methods and fully automatic methods. For the manual segmentation methods, radiologists use the multi-modality data, provided by the medical images along with information pertaining to both the anatomy and the physiology. This kind of segmentation needs the radiologist to manually draw the areas and regions of tumour carefully by going through slices of CT/MRI scans. Therefore, manual segmentation is a tedious, radiologist dependent task, resulting in variable outcomes. However, manual segmentations are useful in the evaluation process of the results obtained from semi-automatic and fully automatic methods.

For semi-automatic methods for image segmentation-based cancer treatment, three processes are usually needed: initialization, intervention response or feedback response and evaluation of results (110). Initialization is usually performed through the process of defining a Region of Interest (ROI), which contains the approximate region of tumor, enabling the automatic algorithm procedure. Thereafter, machine learning algorithms can be used to obtain an optimal result during the process by providing adjustments based on the feedback received in response. Users, such as doctors and physicists can also assess the results and alter or repeat the process if the results are unsatisfactory. The semi-automatic segmentation method called Tumor Cut requires the user to outline the diameter (maximum) of the tumor on medical images (110). Post initialization, a cellular automaton (CA) based seeded tumor segmentation method is run for two steps, firstly applying the algorithm to each imaging modality individually (e.g., T1, T2, and FLAIR), and then obtaining the resultant tumor volume through combining the results. An improved Tumour-Cut semi-automatic method is proposed to use a classification approach where the segmentation problem was reformed into a classification problem (111). Machine learning classification methods for tumour segmentation require large amounts of data in the form of imaging scans (with known ground truth) from numerous subjects to train on which requires intensity bias and noise correction. Alternatively, we can use a subset of voxels from each tissue type. For these selected voxels, the intensity values along with spatial coordinates as features are extracted by the algorithm, and a support vector machine (SVM) is trained to classify all the voxels of the same input image to their respective tissue type (111). Semi-automatic segmentation-based cancer diagnosis consumes less time than manual methods and can obtain efficient results, however, semi-automatic tumor segmentation methods are still prone to intra and inter user variability. Therefore, the recent tumor segmentation research majorly focuses on fully automatic methods.

User interaction is not required in fully automatic tumor segmentation methods. The automatic segmentation is very challenging for some type of tumour, e.g., gliomas. Tumor bearing imaging data is a 3D data where tumor shapes, size and location vary greatly among patients (112). In addition, the tumor boundaries are usually unclear and irregular with discontinuities, posing great challenge especially against traditional edge-based methods (113). For example, brain tumor MRI data obtained from clinical scans or synthetic databases are inherently complex. The MRI machines and protocols used for data collection vary dramatically resulting in intensity biases and other variations for each image in the dataset (114). The need for several modalities to effectively segment tumor sub-regions even adds complexity. Even though the manual and semi-automatic classification methods reported noteworthy performances, novel fully automatic tumor segmentation techniques based on deep learning algorithms are also emerging with exemplary results. Many studies have applied AI to the field of tumour diagnosis. **Table 3** summarizes recent AI-based tumour diagnosis techniques and reviews several tumour types which are suitable for radiotherapy, such as brain tumour, head and neck cancer, esophageal cancer, lung cancer, and metastasis lymph node. Technical features and method accuracy have been investigated for comparison and improvisation. 

**Table 3:** Recent AI-based tumour segmentation techniques.

Method	Technical features	Tumour type	Accuracy
Tchoketch et al. (112)	Gaussian mixture model, Fuzzy C-Means, active contour, wavelet transform and entropy segmentation methods, without the need to any human interaction and prior knowledge for training phases as supervised methodologies in clinical applications.	Brian tumour	69%
Maharjan et al. (113)	Extreme learning machine local receptive fields (ELM-LRF) consisting of convolutional layers and pooling layers and modified softmax loss function.	Brain tumour	Not Applicable
Ali Shah Tirmzi et al. (114)	Experimental work incorporating modified GA, along with SVM learning mechanism on MR brain image.	Brain tumour	98.56%
Abdel-Gawad et al. (115)	Balance contrast enhancement technique (BCET) is used to improve the image features to provide better characteristics of medical images. The proposed GA edge detection method is then employed, with the appropriate training dataset, to detect the fine edges. A comparative analysis is performed on the number of MR scan images.	Brain tumour	99.61%
Kaur et al. (116)	A new feature named density measure for the classification of the LG and HG glioma tumours using the Hilbert transformation technique.	Brain tumour	100%
Dahab et al. (117)	Modified image segmentation techniques on MRI scan images to detect brain tumours; probabilistic neural network (PNN) model based LVQ with image and data analysis and manipulation techniques to carry out an automated brain tumour classification using MRI-scans.	Brain tumour	100%
M.Y. Bhanumurthy, K. Anne (118)	Feature extraction, classification, segmentation and neuro-fuzzy classifier.	Brain tumour	95.65%
Shrasthta Chauhan, Er. Neha Sharma (119)	Histogram thresholding and artificial neural network techniques.	Brain tumour	Not Applicable
T. Chithambaram, K. Perumal (120)	Edge detection and artificial neural network techniques.	Brain tumour	98%
Hollon, Todd C. et al. (121)	Combination of stimulated Raman histology, a label-free optical imaging method and deep convolutional neural networks (CNNs).	Brain tumour	94.6%
M. RajatMehrotra et al. (122)	Deep learning pretrained models includes AlexNet, GoogLeNet, ResNet50, ResNet101, SqueezeNet by using MR images of BT and applied TL on given dataset.	Brain tumour	99.04%
Adel S. Assiri et al. (123)	Ensemble classification (simple logistic regression learning, support vector machine learning with stochastic gradient descent optimization and multilayer perceptron network).	Breast tumour	99.42%
Gauri P. Anandgaonkar, Ganesh S.Sable (124)	Fuzzy C-Means.	Brain tumour	Not Applicable
Yasmeen M. George et al. (125)	Classification models namely multilayer perceptron (MLP) using back-propagation algorithm, probabilistic neural networks (PNN), learning vector quantization (LVQ) and support vector machine (SVM).	Breast cancer	Not Applicable
Cardenas, C.E. et al. (126)	A deep learning algorithm based on deep auto-encoders is used to identify physician contouring patterns.	Head and Neck cancer	93%
Lin, L. et al. (127)	A three-dimensional convolutional neural network is applied for training (818 cases) and validation MRI data sets to construct the AI tool, which was tested in 203 independent MRI data sets.	Head and Neck cancer	88.7%
Guo, Z. et al. (128)	A DenseNet framework based on 3D convolution with dense connections which enables better information propagation and takes full advantage of the features extracted from multi-modality input images.	Head and Neck cancer	Not Applicable
Tang, H. et al. (129)	A deep convolution neural network-based method to automatically delineate OARs in head and neck cancers.	Head and Neck cancer	80.43%
Guo, D. et al. (130)	A novel stratified learning framework to segment OARs, called (SOARS). SOARS divides OARs into three levels, i.e. anchor, mid-level, and small & hard (S&H). Neural architecture search (NAS) is also to automatically search the optimal architecture for each category.	Head and Neck cancer	82.4%
Yousefi, S. et al. (131, 132)	A DenseNet-based end-to-end approach to analyse the contrast similarity between esophageal GTV and its neighbouring tissues in CT scans.	Esophageal cancer	Not Applicable
Jin, D., et al. (133, 134)	Progressive semantically nested network (PSNN) model, is proposed to incorporate joint RTCT and PET information for accurate esophageal GTV segmentation.	Esophageal cancer	82.6%
Hansen, S. et al. (135)	An unsupervised learning based supervoxel clustering framework for lung tumor segmentation in hybrid PET/MRI.	Lung cancer	78.9%
Tan, J. et al. (136)	A GAN-based architecture with a novel loss function based on the Earth Mover distance for lung segmentation.	Lung cancer	93.8%
Barbu, A. et al. (137)	A robust learning-based method for automatic detection and segmentation of solid lymph nodes from CT data.	Metastasis lymph node	83.0%
Zhu, Z. et al. (138)	A distance-based gating strategy in a multi-task framework is proposed to divide the underlying Lymph Node Gross Tumor Volume distributions into “tumor-proximal” and “tumor-distal” categories, and a shared encoder and two separate decoders are adopted to detect and segment two categories.	Metastasis lymph node	78.2%
Chao, C.H. et al. (139)	Graph neural networks (GNNs) is used to model this inter-lymph nodes relationship, and 3D convolutional neural network (CNN) is used to extract lymph node gross tumor volume instance-wise appearance features from CT.	Metastasis lymph node	85%

## 5 Challenges and Future Prospect

### 5.1 Robust Calibration for Biomarkers

Current quantitative imaging approaches have larger voxels and worse signal-to-noise-ratio (SNR) compared to other clinical imaging techniques, since quantitative imaging requires one or multiple quantitative parameters for each voxel (92). For dynamic contrast-enhanced MRI/CT, the acquisition of multiple images is required (1). Therefore, target delineation and dose painting on functional images rely more on the values of parameters of biomarkers compared to other clinical imaging. As was reviewed in Section 2, the main limitation of functional imaging lies in the uncertainties related to the relationship between biomarker and their corresponding parameters. Further studies involving more robust calibration are needed to propose a more robust relationship. One alternative way is to use the heterogeneity of the biomarkers rather than the absolute value of biomarker, which can reduce the impact the uncertainties of the biomarkers (76). For tumor types with high tumor heterogeneity, such as GBM, it makes more sense to consider the biomarker parameters of voxel level instead of the mean value of a whole tumor. Ideally, dose painting prescriptions should be based on dose response data which can be observed from multiple functional imaging methods (108).

### 5.2 AI-Based Biomarkers Quantification

At present, the interest of imaging biomarkers is not only due to the state-of-the-art treatment development and personalized medicine but also due to the recent application of artificial intelligence (AI) algorithms in image processing (140).

AI techniques can help construct image-based biomarkers which can provide a comprehensive view of the entire extent of the tumor and can capture regional tumor heterogeneity (141–143). Since biomarkers are used in a non-invasive (or mildly invasive) way, AI techniques, such as convolutional neural networks (CNN) can facilitate revealing tumor phenotype related to prognosis and monitoring the development and progression of the disease or its response to radiotherapy. Traditionally, CNN-based medical image processing has been used to segment the organ and tumor (140). In addition, it is necessary to explore other kinds of biomarkers (such as ADC, SUV, NAA) instead of tumour size and volume only. However, the quantification of these biomarkers has been difficult. Therefore, by defining biomarkers as a function of a large number of image descriptors (features) extracted from an image, we can employ machine learning or deep learning techniques to analyse these image features. The most discriminatory subset of features can be found to achieve the best performance for tasks such as result prediction (144–146). Savadjiev et al. presented a novel approach which firstly uses a segmentation-specific CNN to narrow the analysis only to the needed organ and tumor area in the image. Then, the relevant radiomic features will be transferred to another neural network model to classify tumor phenotype (140).

However, use of AI to analyse deep radiomics features has several concerns. Firstly, the theoretical analysis of deep radiomic features extraction are not easily interpretable due to the complexity of deep learning models. This is because a deep neural network requires a large number of complex but weak regularities in the data, which has highly complex and non-linear interactions between multiple network nodes and layers (147). In addition, a deep neural network usually contains millions of parameters, which is also a difficult task, involving sufficient compute power, patient data, and reproducible network models (140). Therefore, interests in building customised neural network models used for different medical imaging-based biomarkers have risen, through which a tremendous potential for biomarker qualification is expected.

### 5.3 Quality of Image Registration

Image registration is the process of geometrically aligning two or more images of the same scene taken at different times, from different viewpoints, and/or by different sensors (148).

Since current radiotherapy treatment planning systems have to create a treatment plan on the images produced by a radiation simulator. Most of the simulators are CT-based, therefore, they can only produce anatomical imaging. Properties of functional imaging as mentioned in this article can be employed by image registration to the anatomical imaging. Thereafter, the dose painting can be implemented to deliver more doses to the high-risk area of tumour defined by the functional imaging. As mentioned in Sections 2 and 3, the high quality of registration is important when using functional imaging to perform the dose painting. For DPBC, the uncertainties of registration can be reduced by adding margins to the high-risk area. However, DPBN features the voxel-level analysis and is more sensitive to uncertainty arising in image registration (92). Most treatment planning systems support image registration and fusion to allow the use of multimodality and time-series image data and even anatomical atlases to assist in target volume and normal tissue delineation. Image registration is also needed to assess dose variation during treatment course and enable adaptive radiotherapy, as will be discussed in Section 5.4.

Medical image registration is a broad topic which can be grouped from various perspectives. From an input image point of view, registration methods can be divided into unimodal, multimodal, interpatient, intra-patient (e.g., same- or different-day) registration. From a deformation model point of view, registration methods can be divided in to rigid, affine and deformable methods. From a region of interest (ROI) perspective, registration methods can be grouped according to anatomical sites such as brain, lung registration. From an image pair dimension perspective, registration methods can be divided into 3D to 3D, 3D to 2D and 2D to 2D/3D. There was a survey summarizing algorithms of conventional image registration (148). The Therapy Physics Committee of the American Association of Physicists in Medicine commissioned Task Group 132 to review current approaches and solutions for image registration (both rigid and deformable) in radiotherapy and to provide recommendations for quality assurance and quality control of these clinical processes (149). However, there is no standard mathematical formalism to perform registration, because noise, distortion and complex anatomical variations can occur. In addition, validation of the software systems performance is complicated due to the lack of documentation available from commercial systems. Recently, many researchers studied AI-based image registration. Compared with the conventional image registration, performing registration with AI can be more accurate and generally order of magnitude faster. Fu et al. summarized the details of the latest developments, challenges and trends in AI-based medical image registration methods (150). It is foreseeable that AI will continue to play an important role in this field.

### 5.4 Dose Painting for Adaptative RT

Most RT plans remain “unchanged”. Once the plans are initialized, they are carried out until the end of treatment, along with minimal modifications on top of the original plans (151). RT treatments are usually delivered for the period of several weeks, which is a long and arduous process. In most cases, the tumour size, location and microscopic status (e.g., metabolism and hypoxia) will change, thereby resulting in long-term and even life-long consequences on patients’ life quality after treatment. To address such challenges, the adaptive RT has been developed recently, where interests in functional image-based dose painting have been growing.

**Figure 3:**
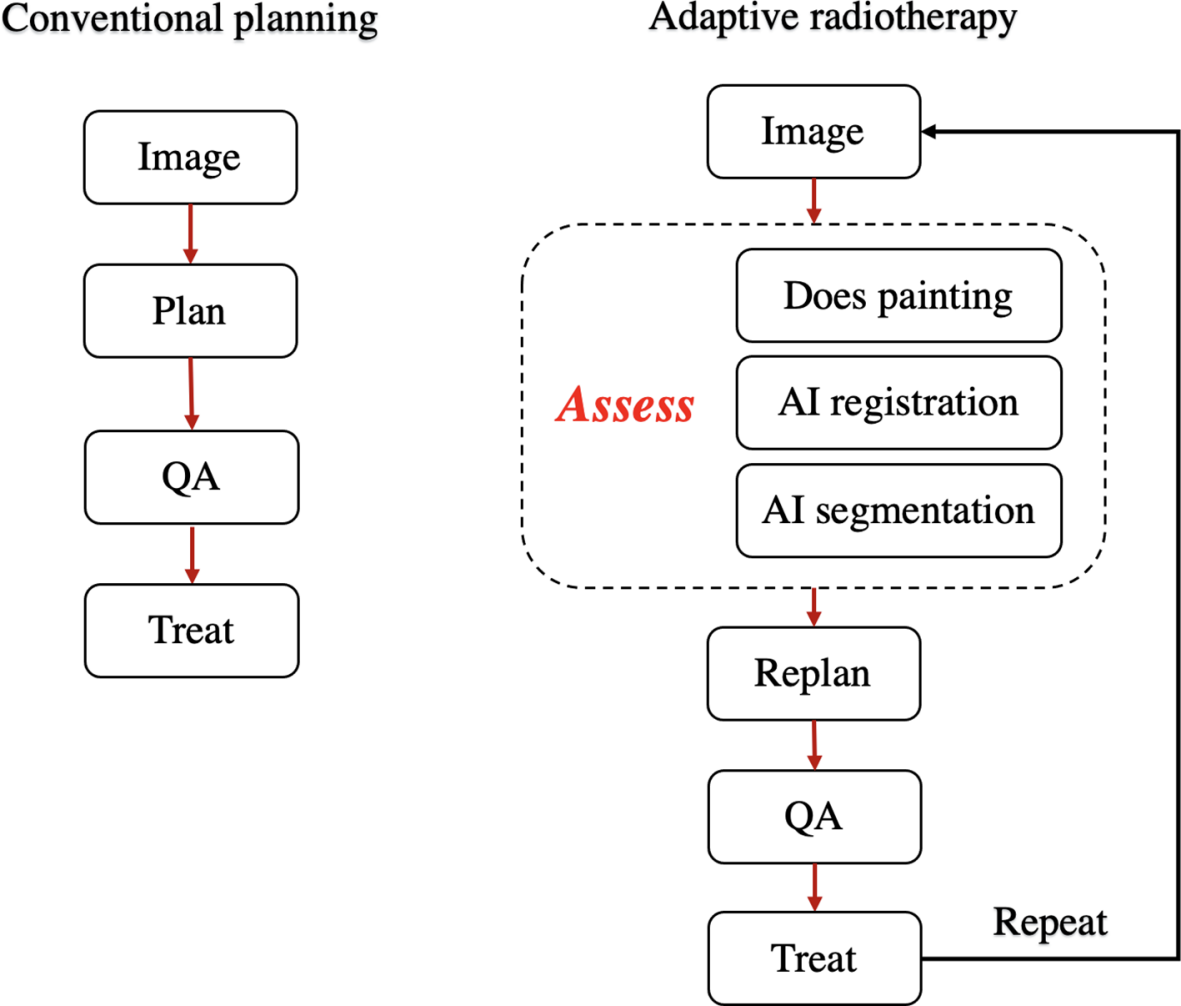
Workflow of conventional and adaptive radiation therapy processes.

Adaptive radiotherapy is a process to control for anatomical and functional variation over the treatment course (126). **Figure 3** shows the workflow of the conventional and adaptive radiotherapy. Both conventional and adaptive radiotherapy require four key technologies: imaging, planning, quality assurance and treatment. Versus conventional radiotherapy workflow, adaptive radiotherapy additionally requires assessment (126). Assessment is the process by which imaging is used to decide whether to adapt the plan or not. This process can range from a manual evaluation to highly automated review of cumulative dose. Therefore, in this step we can embed functional imaging to evaluate the functional variation and re-prescribe dose, employ AI to automatically deline targets and organ at risk, and perform AI-based the image registration. Many studies have discussed the potential of functional images for a more personalized RT planning, however, how to incorporate such quantitative functional imaging into adaptative RT has been barely investigated. For example, since functional imaging can define easy-to-control and hard-to-control areas in the tumor before a treatment, assume that patients take the same functional imaging after a period (e.g., 2 weeks) of radiotherapy, changes in biomarkers can be measured, indicating radio sensitivity and radio resistance after serval fractions of radiotherapy. Adaptative RT can be therefore realisable.

### 5.5 Alleviation of Uncertainties

The field of radiation oncology has been used to address different sources of uncertainties during patient planning, including range and setup uncertainties (152), organ movements (153) and clinical target volume (CTV) definition (154). Consider that the implementation of dose painting depends on the quality of data obtained *via* biomarkers. The interpretation of such data inevitably introduces some uncertainties. Therefore, it is foreseen that potential mathematical tools to solve uncertainties, e.g., partially observable Markov decision processes (POMDP), imperfect state information (ISI) and adjustable robust optimization (ARO) might be necessitated.

## 6 Conclusion

In this article, we reviewed the state-of-the-art functional imaging techniques which facilitates the development of dose painting. Dose painting by contour and dose painting by numbers are discussed in detail, respectively, along with a summary of their advantages and disadvantages. Finally, we conclude some existing challenges and provide corresponding possible research directions. Efforts to develop image biomarkers for tumor characterization have been ongoing for decades. Using DPBC and DPBN could better consider the heterology than the current uniform dose distribution in tumour area. Therefore, they are beneficial for circumventing local recurrence and get better tumour control probability. It is necessary to establish strict pipelines and reproducible workflow, which make DPBC and DPBN feasible and practical in the clinic. In the future, combing the state-of-the-art AI techniques with advanced functional imaging approaches can open up possibilities to conduct new avenues of research in biomarkers discovery to improve cancer diagnosis and treatment.

## Author Contributions

HL: study design. HW and YP: data collection. YP: data interpretation and writing. All authors contributed to the article and approved the submitted version.

## Conflict of Interest

The authors declare that the research was conducted in the absence of any commercial or financial relationships that could be construed as a potential conflict of interest.

## Publisher’s Note

All claims expressed in this article are solely those of the authors and do not necessarily represent those of their affiliated organizations, or those of the publisher, the editors and the reviewers. Any product that may be evaluated in this article, or claim that may be made by its manufacturer, is not guaranteed or endorsed by the publisher.
